# Neurofilament Levels in Dendritic Spines Associate with Synaptic Status

**DOI:** 10.3390/cells12060909

**Published:** 2023-03-15

**Authors:** Clara-Marie Gürth, Maria Augusta do Rego Barros Fernandes Lima, Victor Macarrón Palacios, Angel Rafael Cereceda Delgado, Jasmine Hubrich, Elisa D’Este

**Affiliations:** 1 Department of Optical Nanoscopy, Max Planck Institute for Medical Research, 69120 Heidelberg, Germany; clara-marie.guerth@mr.mpg.de (C.-M.G.); maria.lima@mr.mpg.de (M.A.d.R.B.F.L.); victor.macarron@mr.mpg.de (V.M.P.); angel.cereceda@mr.mpg.de (A.R.C.D.); jasmine.hubrich@mr.mpg.de (J.H.); 2 Department of NanoBiophotonics, Max Planck Institute for Multidisciplinary Sciences, 37077 Göttingen, Germany; 3 Optical Microscopy Facility, Max Planck Institute for Medical Research, 69120 Heidelberg, Germany

**Keywords:** neurofilaments, synapses, dendritic spines, STED nanoscopy

## Abstract

Neurofilaments are one of the main cytoskeletal components in neurons; they can be found in the form of oligomers at pre- and postsynapses. How their presence is regulated at the postsynapse remains largely unclear. Here we systematically quantified, by immunolabeling, the occurrence of the neurofilament isoform triplet neurofilament light (NFL), medium (NFM), and heavy (NFH) at the postsynapse using STED nanoscopy together with markers of synaptic strength and activity. Our data show that, within dendritic spines, neurofilament isoforms rarely colocalize with each other and that they are present to different extents, with NFL being the most abundant isoform. The amount of the three isoforms correlates with markers of postsynaptic strength and presynaptic activity to varying degrees: NFL shows the highest correlation to both synaptic traits, suggesting its involvement in synaptic response, while NFM exhibits the lowest correlations. By quantifying the presence of neurofilaments at the postsynapse within the context of the synaptic status, this work sheds new light on the regulation of synaptic neurofilaments and their possible contribution to synaptopathies.

## 1. Introduction

Neurofilaments (NFs) are among the main components of the cytoskeleton in neurons. They are heteropolymers of NF-light (NFL), NF-medium (NFM), and NF-heavy (NFH), and can further incorporate into their backbone either α-internexin or peripherin in the central or peripheral nervous system, respectively [1]. The NFs’ structure consists of a globular head, a conserved central rod region important for polymerization, and a tail of variable length at the C-terminus. The long tails of NFM and NFH are enriched in phosphorylation sites that protrude into the periphery of the filament (reviewed in [2,3,4]). Variability in the subunit stoichiometry and the dynamic phosphorylation landscape indicate that NFs have a high potential for being finely regulated [5]. Indeed, these features are differentially modulated based on the cell type, developmental stage, and subcellular compartment [2,3].

Traditionally, NFs have been considered as structural components in the axon, albeit also present in dendrites and synaptosomes [6,7,8,9]. Although the role of NFs in signal transduction was proposed decades ago [10,11,12], a link between NF function in synapses and synaptic disorders has started to emerge only in the last years [13,14,15,16]. Indeed, NFs were identified at pre- and postsynaptic sites of excitatory and inhibitory synapses in the form of short 9–10 nm long filaments corresponding to oligomeric structures [17,18]. However, immunogold electron microscopy showed that all subunits appear more concentrated in the postsynaptic compartment than at the presynapse. Interestingly, the degree of phosphorylation of NF C-terminal tails was lower for NFM and higher for NFH, when comparing immunoblots on hippocampal synaptosomes to the overall NF population [18]. Further evidence for a specific role of NFs at synapses is that long-term potentiation (LTP) and depression (LTD) induce a site-specific NFL phosphorylation in apical dendrites of pyramidal neurons [19,20]. Additionally, alterations of NFs phosphorylation levels are reported in several neuropsychiatric disorders (reviewed in [13]). Deletion of NFH was shown to depress LTP in the hippocampus without altering spines morphology, which, on the other hand, is affected in NFL knockout animals [18,21]. Those animals show a dysfunctional hippocampus-dependent spatial memory and exhibit a schizophrenia-like behavior [18]. These phenotypes are linked to the role of both NFL and α-internexin in regulating the localization of N-methyl-D-aspartate (NMDA) receptor subunits. Lastly, NFM functionally interacts with dopamine D1-receptors. Hence, direct evidence for the involvement of NFs in the regulation of synaptic functions exists [16,18,21,22,23,24]. However, despite a growing body of evidence pointing at NFs as important synaptic components, whether and how their presence in the dendritic spine correlates with the synaptic status, and in particular the presynaptic activity and the postsynaptic strength, is unclear. Answering this question would reveal whether NFs play a role in the short-term response to synaptic activity or in the structural rearrangements occurring during LTP.

To clarify the regulation of the NFs presence in postsynaptic compartments, we used quantitative multicolor super resolution stimulated emission depletion (STED) nanoscopy of the NF triplet NFL, NFM, or NFH in combination with proxies of either postsynaptic strength (homer) or of presynaptic activity (synaptotagmin-1, Syt1, live-cell uptake [25,26]) on cultured hippocampal neurons. We found that NFL is the most abundant isoform in dendritic spines. Overall, the amounts of NF isoforms correlated with both homer and Syt1 signal to varying extents, with NFL showing the highest and NFM the lowest correlation, suggesting that their occurrence might be influenced by the postsynaptic strength and activity.

## 2. Materials and Methods

### 2.1. Preparation of Neuronal Cultures

All procedures were performed in accordance with the German Animal Welfare Act (Tierschutzgesetz der Bundesrepublik Deutschland, TierSchG) and the Animal Welfare Laboratory Animal Ordinate (Tierschutz-Versuchstierverordnung, TierSchVersV), according to which no specific ethical authorization or notification is required. The sacrificing of P0–P2 rats was supervised by animal welfare officers of the Max Planck Institute for Medical Research (MPImF) and conducted and documented according to the guidelines of the TierSchG (permit number assigned by the MPImF: MPI/T-35/18).

Primary hippocampal cultures were prepared from dissociated tissue of P0-P2 postnatal wild-type Wistar rats of either sex (Janvier-Labs, Le Genest-Saint-Isle, France), as described previously in [26]. Briefly, dissected hippocampal tissue was digested with 0.25% trypsin for 20 min at 37 °C, dissociated, and maintained in Neurobasal supplemented with 2% B27, 1% GlutaMAX and 1% penicillin/streptomycin (all from Gibco, Thermo Fisher Scientific, Waltham, MA, USA). Cells were seeded at a concentration of 110,000/well in 12-well plates on ∅ 18 mm glass coverslips coated with 0.1 mg/mL poly-ornithine (Sigma-Aldrich/Merck, Darmstadt, Germany) and 1 µg/mL laminin (Corning, New York, NW, USA). Medium was changed to fresh supplemented Neurobasal 1–2 h after seeding and cultures were maintained in an incubator (37 °C, 5% CO_2_, 95% rH) without inhibition of glial cell growth by AraC.

### 2.2. Recombinant AAV-hSyn-EGFP Production

To obtain recombinant adeno-associated viral AAV-hSyn-EGFP vectors of mixed serotype 1 and 2, HEK 293FT cells were transfected via TransIT-293 reagent (Mirus Bio LLC, Madison, WI, USA, cat. 2704) in a molar ratio of 1:1:1 with a total of 15 µg of the following plasmids: pFdelta6 adenovirus helper plasmid (Addgene Watertown, MA, USA, #112867, a gift from James M. Wilson), pAAV 2/1 plasmid (Addgene #112862, a gift from James M. Wilson) containing capsid protein sequences for serotype 1 and replication protein sequences for serotype 2, and the donor plasmid pAAV-hSyn-EGFP (Addgene #50465, a gift from Bryan Roth) containing the recombinant expression cassette flanked by AAV packing signals ITRs. After 2 days, the cells were resuspended in lysis buffer (NaCl 150 mM, Tris-HCl 50 mM; pH 8.5), cracked by 3 freeze-thaw cycles (−80 °C for 20 min, 37 °C for 10 min) and treated with 1 μL/mL DNAseI (Thermo Fisher Scientific, 1 unit/μL; 37 °C for 30 min). The lysate was centrifuged (900× *g* for 10 min and 2400× *g* for 3 min) to remove all cell fragments, and the supernatant was centrifuged in a third round to pellet the virus particles (48,000× *g* for 2 h at 4 °C). The pellet was resuspended in 150 μL sterile PBS (pH 7.4) and aliquots were stored at −80 °C. Virus titer (6 × 10^11^–1.2 × 10^13^ GC/mL) was measured via qPCR following the respective Addgene protocol [27].

### 2.3. Sample Preparation and Immunostaining

Cultured neurons were transduced at DIV 7–8 for volume labeling with the adeno-associated viral vector AAV-hSyn-EGFP by adding 0.25–1 µL of the viral preparation directly to the medium and kept in culture until use.

Turnover of synaptic vesicles in mature cultures (DIV 22-25) was determined by live labeling with Atto647N-labeled mouse antibody against the luminal domain of synaptotagmin-1 (Synaptic Systems, Göttingen, Germany, cat. 105 311AT1, 1:500 in culture medium) for 1 h, as previously described [25,26]. Samples were then washed three times in prewarmed ACSF (126 mM NaCl, 2.5 mM KCl, 2.5 mM CaCl_2_, 1.3 mM MgCl_2_, with 30 mM Glucose, 27 mM HEPES, all from Sigma-Aldrich/Merck) before fixing.

On the day of immunolabeling, mature neuronal cultures were fixed in 4% PFA in PBS, pH 7.4, quenched with quenching buffer (PBS, 100 mM glycine, 100 mM ammonium chloride), permeabilized for 5 min in 0.1% Triton X-100 in PBS, and blocked with 1% BSA in PBS for 1 h. Samples were then incubated for 1 h at room temperature with primary antibodies and secondary antibodies in PBS, each followed by five washes in PBS. Primary antibodies used were: homer1 guinea pig (Synaptic Systems, cat. 160 004, 1:500 dilution), ankyrin G guinea pig (Synaptic Systems cat. 386 005, 1:1000 dilution), ankyrin G rabbit (Synaptic Systems cat. 386 003, 1:1000 dilution, Figure S4, channel not shown), neurofilament-L rabbit (Synaptic Systems, cat. 171 002, AA 1 to 284 from UniProt Id: P07196, 1:200 dilution), neurofilament-L mouse (Sigma-Aldrich/Merck, ascites fluid, cat. N5139, 1500 dilution), neurofilament-M rabbit (Synaptic Systems, cat. 171 203, AA 763 to 848 from UniProt Id: P08553, 1:200 dilution), neurofilament-H rabbit (Synaptic Systems, cat. 171 102, AA 998 to 1097 from mouse UniProt Id: P19246, 1:200 dilution), and neurofilament-H mouse (Synaptic Systems, cat. 171 111, AA 998 to 1097 from mouse UniProt Id: P19246, 1:200 dilution). For colocalization, samples NF primary antibodies were used at 1:400 dilution. Secondary antibodies used were STAR 635P anti-guinea pig (Abberior, Göttingen, Germany, cat. 2-0112-007-1, 1:100 dilution, 1:200), Alexa Fluor 594 anti-rabbit (Thermo Fisher Scientific, cat. A-21207, 1:100 dilution), STAR 580 anti-mouse (Abberior, cat. ST580-1001–500 µg, 1:200 dilution), STAR 635P anti-rabbit (Abberior, cat. 2-0022-052-9, 1:200 dilution), Alexa Fluor 405 anti-guinea pig (Abcam, Cambridge, United Kingdom, ab175678, 1:1000 dilution), and Alexa Fluor 405 anti-rabbit (Thermo Fisher Scientific, cat. A 31556, 1:1000 dilution for experiments shown in Figure S4). Samples were then embedded in Mowiol^®^ (Sigma-Aldrich/Merck, cat. 81318) supplemented with DABCO (Sigma-Aldrich/Merck, cat. 290734) and cured for at least 1 h at room temperature before imaging. Imaging was performed within 1 week of sample preparation.

### 2.4. Confocal and STED Imaging

Samples were imaged using an Abberior Expert Line Microscope (Abberior Instruments GmbH, Göttingen, Germany) on a motorized inverted microscope IX83 (Olympus, Tokyo, Japan) and equipped with pulsed STED lines at 775 nm and 595 nm, excitation lasers at 355 nm, 405 nm, 485 nm, 561 nm, and 640 nm, and spectral detection. Spectral detection was performed with avalanche photodiodes (APD) and detection windows were set to 650–725 nm, 600–630 nm, 505–540 nm, and 420–475 nm to detect Atto647N/STAR 635P, Alexa Fluor 594/STAR 580, EGFP, and Alexa Fluor 405, respectively. Images were acquired with a 100×/1.4 NA oil immersion lens, 30 nm pixel size, and pinhole to 100 µm (1 A.U.). Laser powers and dwell times were kept consistent during the entire experiment.

### 2.5. Image Processing and Analysis

Images shown in figures were visualized and processed with Imspector (Abberior Instruments GmbH), Fiji (version 1.53f51, [28]) and MATLAB 2021b (MathWorks, Natick, MA, USA). Images are shown as smoothed data with a low pass Gaussian filter and 1–5% background subtraction. Brightness was adjusted uniformly throughout the images. Manual segmentation of axons and spines were performed in Fiji. The axons and spines were manually segmented based on their morphology as visualized by EGFP volume labeling and axon initial segment (AIS) marker ankyrin G [29,30]. Axons were identified by following ankyrin G-positive processes and, in the absence of distal ankyrin G signal, as thin and smooth neurites without any protrusion (<1–2 µm in thickness). Broader and irregular processes were considered as dendrites. Ambiguous processes were not considered. Spines were identified as protrusions from the dendritic shaft and no cutoff to spine size was applied (Figure S2). Spines crossed by bright axonal NF signals were excluded from the analysis.

In addition to the spine segmentation, a second mask was generated by applying an automatic Otsu’s threshold to STED images of synaptic markers or a 4-counts background threshold for STED images of NFs. Images with very bright spots were saturated to 0.01% their highest intensity. Each image was then masked with the intersection between the defined spine regions and its respective second mask. Afterwards, for each region of interest (ROI), the area and the intensity of the enclosing pixels were computed. The ROI amount was defined as the ROI area times the ROI mean intensity.

For comparing amounts of NF and synaptic markers, we used the standardized data sorted by experimental rounds. The standardization was computed as $z=\frac{x-\mu }{\sigma }$ , where *x* is the input data point, *µ* is the group mean, and σ the group standard deviation. The Spearman’s correlation coefficient was then calculated for each NF isoform versus the synaptic marker after grouping all the corresponding standardized experimental rounds. Spearman’s correlation coefficients were interpreted as suggested in [31] (0.3–0.5 low correlation, 0.5–0.7 moderate correlation).

Colocalization was performed using a custom code written in MATLAB to compute the non-zero Pearson’s correlation coefficient. To quantify the signal of NF isoforms in axons and spines, the mean intensity, defined as the total sum of intensities of non-zero pixels divided by the total number of non-zero pixels in a masked image, was used.

The images from STAR 635P and STAR 580 channels were unmixed with Spectral Unmixing Fiji plug-in (v1.3), using single-color samples as reference. For analysis on the cellular level, the NF signal of the entire field of view was segmented from the background with a binary mask. The mask was generated after saturating 90% of the original confocal image, followed by an 8-pixel median filtering to remove noisy pixels, and by a global Otsu’s threshold. On the spine and axon level, we used the masks generated from the manual segmentation of STED images described above. Thereafter, the respective masks were applied to the original images and the Pearson’s correlation coefficient for non-zero pixels was computed for each pair of NF isoforms to characterize their degree of colocalization. Signal intensities in axons and spines were calculated on the same non-zero pixels included in the respective masks, after offsetting the minimum image intensity to 0.

## 3. Results

### 3.1. NF Isoforms Poorly Colocalize at the Postsynapse

**Figure 1:**
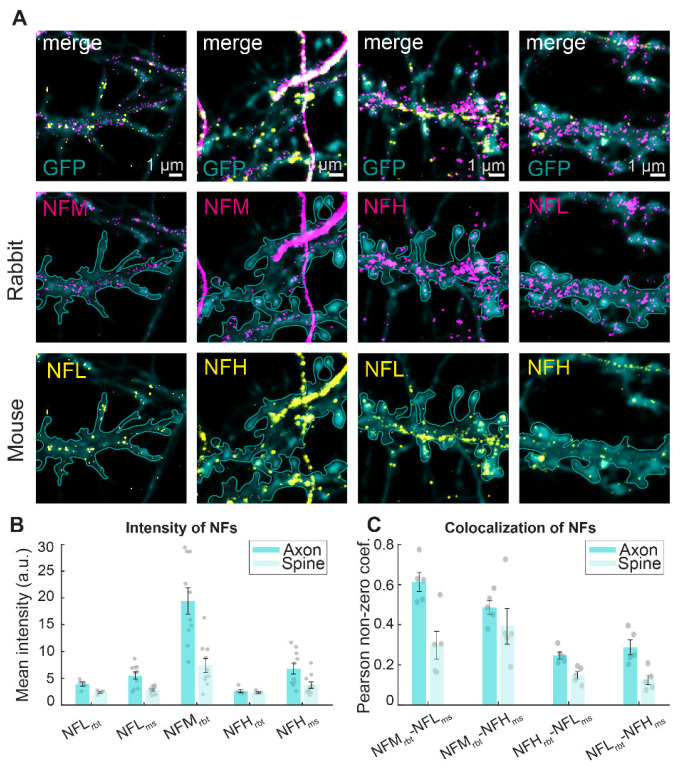
Distribution of NF signal in different neuronal compartments. (**A**) Representative STED images of NF isoforms (magenta for rabbit (rbt) and yellow for mouse (ms)) and confocal GFP volume labeling (cyan) in primary hippocampal neurons. Corresponding large fields of view images can be found in Figure S1. Cyan outline represents spine shapes on dendrites as determined by volume labeling. Scale bars are 1 µm. (**B**) Mean fluorescence intensity of the NF isoforms in axons and dendritic spines. (**C**) Colocalization analysis of NFs in axons and dendrites. Shown is the mean values of Pearson’s correlation coefficient of all pixels with non-zero intensities. Error bars show the standard error of the mean (SEM), points represent average values of individual images. Intensities and colocalization data calculated from 5 images per antibody pair from one neuronal culture (21–26 DIV). Number of spines: NFM_rbt_-NFL_ms_ = 171; NFM_rbt_-NFH_ms_ = 63; NFH_rbt_-NFL_ms_ = 148; NFL_rbt_-NFH_ms_ = 100.

To analyze NFs at the postsynapse while preserving the isoform specificity and spatial resolution, our method of choice was immunofluorescence labeling. Therefore, we selected isoform-specific antibodies against the NF isoforms NFL, NFM, and NFH and tested them in hippocampal neurons. We selected antibodies raised in mouse and rabbit against NFL and NFH, and in rabbit for NFM. All selected antibodies exhibited a strong signal in axons (Figure 1A and Figure S1). However, a signal was also visible in non-axonal regions. High resolution multicolor STED imaging of the NF isoforms in combination with confocal GFP volume labeling and an axon initial segment marker (ankyrin G), through which axons can be unequivocally recognized, showed that the non-axonal signal is generated by immunoreactivity along dendrites and dendritic spines (Figure 1A and Figure S1). To characterize the signal in the different neuronal compartments, axons and dendritic spines were manually segmented based on the morphology of the GFP labeling and the ankyrin G signal (Figure S2) and the mean intensity was measured. Analysis demonstrated that the fluorescence intensity signal along the axon was 1.1 to 2.5 times higher than the signal in dendritic spines (Figure 1B).

Next, we focused on dendritic spines to assess the role of NF isoforms at the postsynapse. To this end, we performed dual-color STED imaging by combining a rabbit and a mouse antibody and using the fluorophore pair STAR 635P and STAR 580. These dyes can be depleted with a common STED depletion line at 775 nm, therefore ensuring intrinsic alignment of the two channels. Pearson correlation coefficient on pixels with non-zero intensity in manually segmented spines showed a low degree of colocalization. Values range from 39% of colocalization of NFM and NFH to 12–15% of colocalization of NFL and NFH. Importantly, in all cases, the colocalization observed in the spines was lower than the colocalization observed along axons (Figure 1C). Furthermore, the low colocalizations cannot be ascribed to chromatic differences, since control experiments in which one antibody isoform (NFM) was labelled with a cocktail of secondary fluorophores carrying an orange and red dye led to a colocalization of 75%. This shows that NF isoforms are present in the postsynaptic compartment, although in lower amount compared to axons and with an overall low degree of colocalization.

### 3.2. NFL Is the Most Represented Isoform in Dendritic Spines

**Figure 2:**
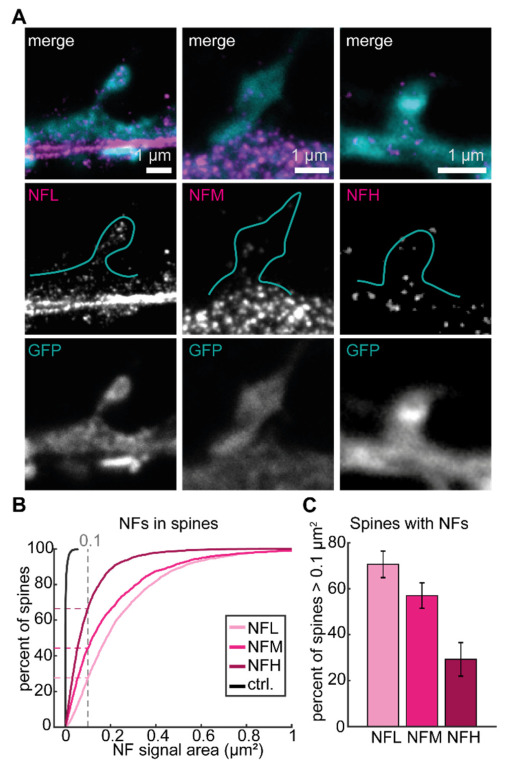
NF isoforms are found at the postsynapse. (**A**) Representative STED images of dendritic spines with NFs (magenta) and volume labeling (confocal, cyan) with NFL (left), NFM (middle), or NFH (right). Spine shape as seen in volume labeling is represented in cyan outline. Scale bars are 1 µm. (**B**) Cumulative distribution of spines containing NFs compared to control without primary antibody labeling. Dashed lines indicate individual values at a 0.1 µm^2^ threshold. (**C**) Percentage of spines containing more than 0.1 µm^2^ of NF signal. Data was calculated from a total of 7115 spines (NFL = 2242, NFM = 1775, NFH = 2342, ctrl = 756) manually segmented from 34–40 images per isoform from 8 independent neuronal cultures (14–36 DIV), 15 images from 3 independent neuronal cultures for ctrl). Error bars show the standard error of the mean (SEM).

To further characterize the presence of NFs at the postsynapse, we next performed comparative STED imaging of either NFL, NFM, or NFH. To minimize the variability of the experiments, we chose primary antibodies raised in rabbit for all isoforms so that the same secondary antibody could be used for all experiments (Figure 2A). The presence of NFs inside dendritic spines was quantified based on the intensity and area of NF signal inside the manually segmented spines, as identified by GFP volume labeling. The cumulative distribution of NF area within individual spines showed that, in most of the spines, the area covered by NFL signal is larger than the area covered by NFM and NFH. Control images with only secondary antibody labeling showed low background signal compared to the NF signal (Figure 2B and Figure S3). Based on the cumulative distribution we tested the presence of the NF signal area with a threshold of 0.1 µm^2^. We observed that around 70% of spines contained more than 0.1 µm^2^ NFL signal per spine whereas this value dropped to 55% and around 30% for NFM and NFH, respectively (Figure 2C). This demonstrates that NF isoforms are present at the postsynapse to varying extents and suggests a distinct role of NFL and NFM or NFH within dendritic spines.

### 3.3. Correlation between Postsynaptic Strenght Marker Homer and NFs in Spines

**Figure 3:**
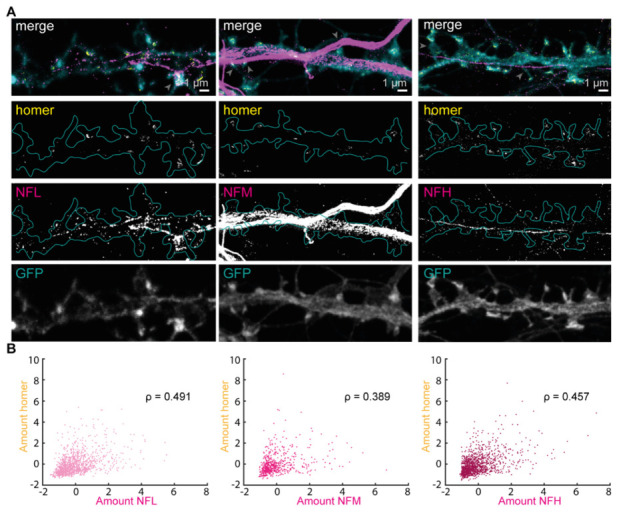
Positive correlation between NFs at the postsynapse and postsynaptic strength. (**A**) Representative dual-color STED images of neurons with homer (yellow), NFs (magenta), and volume labeling (confocal, cyan) with NFL (left), NFM (middle), or NFH (right). Cyan outline represents spine shapes on the dendrite as determined by volume labeling. Scale bars are 1 µm. Only signal above the 4-counts threshold used for data analysis is shown. Gray arrows in merge images point at examples of spines that were not considered for the analysis due to the overlay of strong axonal signal. (**B**) Scatterplot of NF versus homer protein amount ( area ×mean intensity ). ρ indicates Spearman’s correlation coefficient with *p* values for NFL: 1.124 × 10^−72^; NFM: 3.564 × 10^−24^ and NFH: 4.202 × 10^−61^. Data was obtained from 3243 manually segmented spines (1248, 809, and 1186 for NFL, NFM and NFH, respectively) from 19–21 images per isoform and 6 independent neuronal cultures (14–21 DIV), standardized to the mean of each experiment. Amounts represent arbitrary units.

After having quantified the NF content at the postsynapse, we questioned whether the presence of NFs depends on different synaptic traits. First, we tested the connection with postsynaptic strength as described previously [26]. We quantified the STED signal of the postsynaptic scaffolding protein homer, which regulates receptors’ clustering [32], together with the STED signal of either NFL, NFM or NFH in manually segmented spines (Figure 3A). The amount of NF and the amount of homer were determined based on the area and mean intensity of the respective STED signal.

The correlation between these two relative protein amounts was highest for NFL (ρ = 0.491), followed by NFH (ρ = 0.457) and NFM (ρ = 0.389) (Figure 3B). Since NFL is also the most abundant isoform in the spines (Figure 2), we validated this result with the antibody raised in mouse [18]. These experiments confirmed the results obtained with the antibody raised in rabbit and indicate the presence of a moderate correlation (ρ = 0.604) (Figure S4). Therefore, a positive correlation exists between NFs and postsynaptic strength as characterized by homer labeling; this is strongest for NFL. This indicates that spines with a lower homer amount also have a lower amount of NFs, and spines with higher levels of homer also contain more NFs, and NFL in particular.

### 3.4. Postynaptic NFL Exhibits the Strongest Correlation with Presynaptic Vesicle Recycling

**Figure 4:**
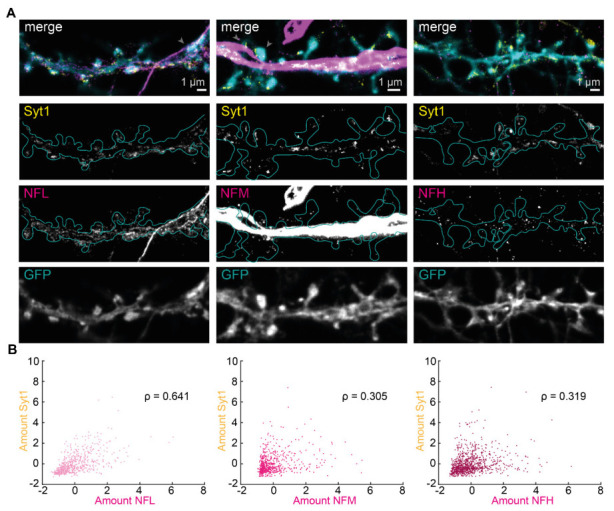
NFL at the postsynapse correlates with synaptic activity. (**A**) Representative dual-color STED images of neurons with Syt1 (yellow), NFs (magenta), and volume labeling (confocal, cyan) with NFL (left), NFM (middle), or NFH (right). Cyan outline represents spine shapes on the dendrite as determined by volume labeling. Scale bars are 1 µm. Only signal above the 4-counts threshold used for data analysis is shown. Gray arrows in merge images point at examples of spines that were not considered for the analysis due to the overlay of strong axonal signal. (**B**) Correlation scatterplot of NF protein amount and amount of labeled Syt1 ( area ×mean intensity ). ρ indicates Spearman’s correlation coefficient with p values for NFL: 2.55 × 10^−75^; NFM: 2.23 × 10^−15^, and NFH: 1.26 × 10^−23^. Data was obtained from 2398 manually segmented spines (686, 779, and 933 for NFL, NFM and NFH, respectively) from 16–18 images and 3 independent neuronal cultures (22–25 DIV), standardized to the mean of each experiment. Amounts represent arbitrary units.

We then questioned whether the presence of NFs in dendritic spines is further regulated by presynaptic activity. We performed activity dependent live-labeling of Syt1, a proxy of synaptic activity, as previously described [25,26]. The used antibody recognizes a luminal epitope of Syt-1, which becomes accessible upon fusion of the synaptic vesicles, and gets internalized during the endocytosis processes. In this way, specific labeling of recycling vesicles is achieved. Live immunolabeling of Syt1 was combined with immunolabeling of the three different NF isoforms as in previous experiments (Figure 4A). We then correlated the amount of NFL, NFM or NFH with the number of actively recycled synaptic vesicles at individual synapses. We observed a moderate correlation between synaptic activity and the presence of NFL (ρ = 0.641), showing that more active synapses have larger amounts of NFL at their postsynaptic site (Figure 4B). Interestingly this correlation was lower for NFM and NFH amounts (ρ = 0.305 and 0.319, respectively). Hence NFL, the NF isoform that is most abundant at the postsynapse, shows the strongest dependence not only to synaptic strength but also to synaptic activity.

## 4. Discussion

NFs belong to the most abundant proteins in neurons and are found in both axonal and dendritic regions. Although several studies suggest other NF functions beyond axonal structural support, and connections with psychiatric disorders are known, their role at the synapse is only beginning to emerge [13,14,15]. In the last decade, the presence of NF oligomers has been reported at postsynaptic sites, where they influence receptor function, LTP induction, and spine morphology [3,13]. In this work, we used quantitative multicolor nanoscopy of the NF triplet to characterize its distribution and identify correlations between its presence and synaptic traits. In particular, we analyzed the correlation between NFL, NFM, and NFH with either a marker of postsynaptic strength (homer) or a marker of presynaptic activity (Syt1) [25,26].

The use of STED nanoscopy in combination with volume labeling enabled us to differentiate the postsynaptic sites from dendritic or axonal shafts with high lateral resolution. However, the possibility exists that a signal coming from overlapping pre-axonal terminals is detected within the same focal volume, deteriorating the specificity of the results. Importantly, the abundance of NF is higher at the postsynapse than at the presynapse [3]. Furthermore, spines overlapping with an obvious bright axonal signal were excluded from the analysis. Therefore, the impact of this technical limitation is minimized in this study. Since spines were identified as protrusions from the dendritic shaft, the results best represent glutamatergic postsynaptic sites located on filopodia, mushroom, or stubby spines.

Our study is based on chemical fixation and antibody labeling. Hence, the results are strongly dependent on the preservation of the structures, the specificity of the used reagents, and their ability to bind to the target structure. The influence of these aspects is commonly validated by using knock-out cells or blocking peptides, which are not always accessible. A complementary approach is live-cell imaging; however, this is extremely challenging in the case of NFs due to the difficulties in tagging them while preserving their function [6]. Nonetheless, the axonal signal resembles the pattern observed by light and electron microscopy, and the punctate signal visualized by STED at postsynapses is in agreement with electron microscopy data showing the presence of oligomers at synaptic sites [6,18]. The capability of the antibodies to bind their target structure is relevant in the case of NFs, since they can be highly phosphorylated and the phosphorylation status of NF at synapses is distinctive compared to the rest of the neuron [13,33]. Hence, the affinity of antibody labeling might differ based on the post-translational modification, and the possibility exists that not all NF molecules are being detected. While this might have an impact on absolute quantification of synaptic NFs, this should only marginally affect the comparative correlation analyses as applied here. Indeed, it can be reasonably assumed that, within one sample, the fraction of detected NF molecules is the same, regardless of the synaptic status.

Analysis of postsynaptic compartments revealed NF signal in the majority of dendritic spines. In these compartments, the area occupied by the fluorescent signal is larger for NFL than NFM and NFH. This difference cannot be ascribed to different optical resolutions, since the same fluorophores and imaging conditions were used and therefore represent a broader distribution of the antibody labeling. The higher abundance of NFL in dendritic spines supports the evidence that this isoform is essential for maintaining spine structure and function [3]. Furthermore, at postsynaptic sites, we observed a poor colocalization of the NF isoforms, suggesting that they do not exclusively assemble in hetero-filaments and supporting the evidence that NF are present as oligomeric structures [18]. The presence of longer filaments with an internexin-rich content cannot be excluded. Together with the demonstration of isoform-specific interaction with receptors at synapses (e.g., NFL and NMDA receptors, NFM, and D1 dopamine receptors [14,16,24]), this data points at isoform-specific functions of NFs within the context of the postsynapses.

To follow up on the hypothesis that different NF isoforms have different functions at the postsynapse, we investigated whether their presence depends on synaptic traits such as synaptic strength or activity. These analyses showed that the postsynaptically less abundant isoforms (NFM and NFH) also show lower correlation with synaptic strength and activity. NFM shows the strongest enrichment in axons compared to spines and has been shown to interact with dopaminergic receptors. Since, in primary hippocampal cultures, the majority of the population is represented by glutamatergic and not dopaminergic neurons [34], the low level of correlation for NFM is unsurprising; it is in agreement with evidence that NFM-null mice exhibit normal neurotransmission and LTP induction [3]. In NFH-null mice, however, LTP is impaired via yet unknown mechanisms. The lower level of correlation between NFH amount and presynaptic activity could therefore suggest its additional involvement in activity independent synaptic processes. Notably, the epitope recognized by the NFH antibody (AA998-1097 of mouse NFH [35]) presents only a single phosphorylation site and therefore it is unlikely that the reduced correlation is due to different posttranslational modifications.

NFL showed a slightly different behavior than NFM or NFH. While the correlation was the highest, though still in a comparable range, for synaptic strength, it was nearly twice as high for synaptic activity. Although this might be partially due to mere changes in spine size, it importantly indicates that more active synapses had higher amounts of NFL present at the postsynapse. This suggests a contribution of NFL to short-term plasticity and immediate response to activity changes, as well as an involvement in long-term structural responses that would eventually result in an increased synaptic strength. Since NFL is known to interact with NMDA receptors via the GluN1 subunits, the correlation between synaptic activity and NFL amount could also demonstrate its direct involvement in the modulation of synaptic signaling [21]. As a cytoskeletal component, the involvement of NFL might include NMDA receptor positioning or more indirect changes mediated by the actin cytoskeleton.

In this study we systematically quantified the presence of NFs at the postsynapse within the context of the synaptic status. Further work will be needed to clarify the behavior of internexin and developmentally regulated intermediate filament isoforms [36], how the presence of NFM and NFH at the postsynapse is regulated, and the mechanisms through which NFL responds to synaptic activity. With respect to the latter aspects, we envision two-color live STED experiments by leveraging on Syt-1 live labeling, minimally invasive NF tagging [6], and event-triggered imaging [37].

## Data Availability

Raw imaging data is made available upon reasonable request.

## Supplementary Materials

The following supporting information can be downloaded at: https://www.mdpi.com/2073-4409/12/6/909/s1, Figure S1: Large field-of-view imaged of NFs signal in neuronal cultures; Figure S2: Manual segmentation of dendritic spines and axons; Figure S3: Intensity quantification of NF and synaptic labeling at manually segmented postsynapses in dual color STED images; Figure S4: Correlation between NFL (mouse antibody) and homer.
