# Supplementary material for: Neurofilament Levels in Dendritic Spines Associate with Synaptic Status
Source: Cells. 2023 Mar 15;12(6):909. doi: 10.3390/cells12060909 (PMC10047839; doi:10.3390/cells12060909)
Supplement: Supplementary file 1 [file cells-12-00909-s001.zip › cells-2210389-supplementary.pdf]

Supplementary information

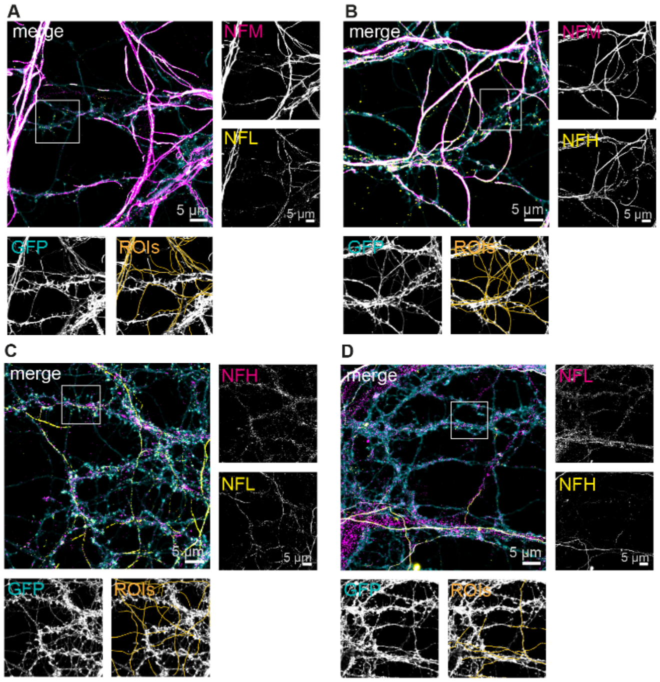

Figure S1. Large field-of-view images of NFs signal in neuronal cultures. Representative STED images of NF isoforms with antibodies raised in rabbit (rbt, magenta) or mouse (ms, yellow) and confocal GFP volume labeling (cyan) in primary hippocampal neurons. Co-staining of (A) NFM (rbt) and NFL (ms), (B) NFM (rbt) and NFH (ms), (C) NFH (rbt) and NFL (ms), (D) NFL (rbt) and NFH (ms). Merged image (left), corresponding single color NF channels (right), and volume labeling and segmented axons (orange, bottom). Samples were additionally stained with ankyrin G (guinea pig, not shown). Scale bars: 5 µm. Boxes indicate regions displayed as close up images in Figure 1.

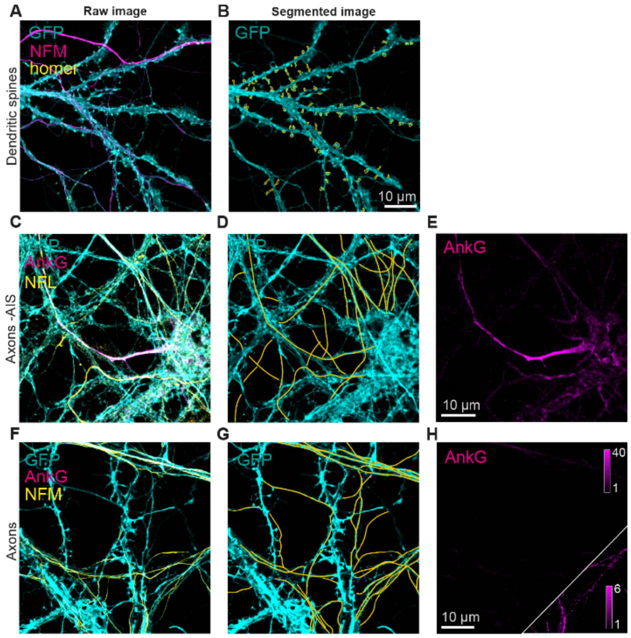

Figure S2. Manual segmentation of dendritic spines and axons. (A) Example of a 3-color image in which NFM (magenta) and homer (yellow) are acquired in STED mode, while the GFP volume labeling (cyan), acquired in confocal mode, is used to manually segment dendritic spines. (B) The outline of the manually segmented spines is overlaid to the GFP channel. (C) Example of axon initial segment (AIS) in a 3-color sample labeled with NFL (rabbit, yellow), GFP volume labeling (cyan), and ankyrin G (guinea pig, magenta). (D) The outline of the manually segmented axons (orange) is overlaid to the GFP channel. (E) Single color ankyrin G signal (1-40 counts). (F) Example of a 4-color image in which NFL (mouse, not displayed) and NFM (rabbit, yellow) are acquired in STED mode, while ankyrin G (magenta, guinea pig) and GFP (cyan) are acquired in confocal mode. For simplicity, only one NF isoform is displayed (same field of view is shown in Figure S1A). (G) The outline of the manually segmented axons (orange) is overlaid to the GFP channel. (H) Single color ankyrin G signal. Upper left corner shown with the same brightness as panel E (1-40 counts), lower right corner shown with linearly increased brightness (1-6 counts). Scale bars: 10 μm.

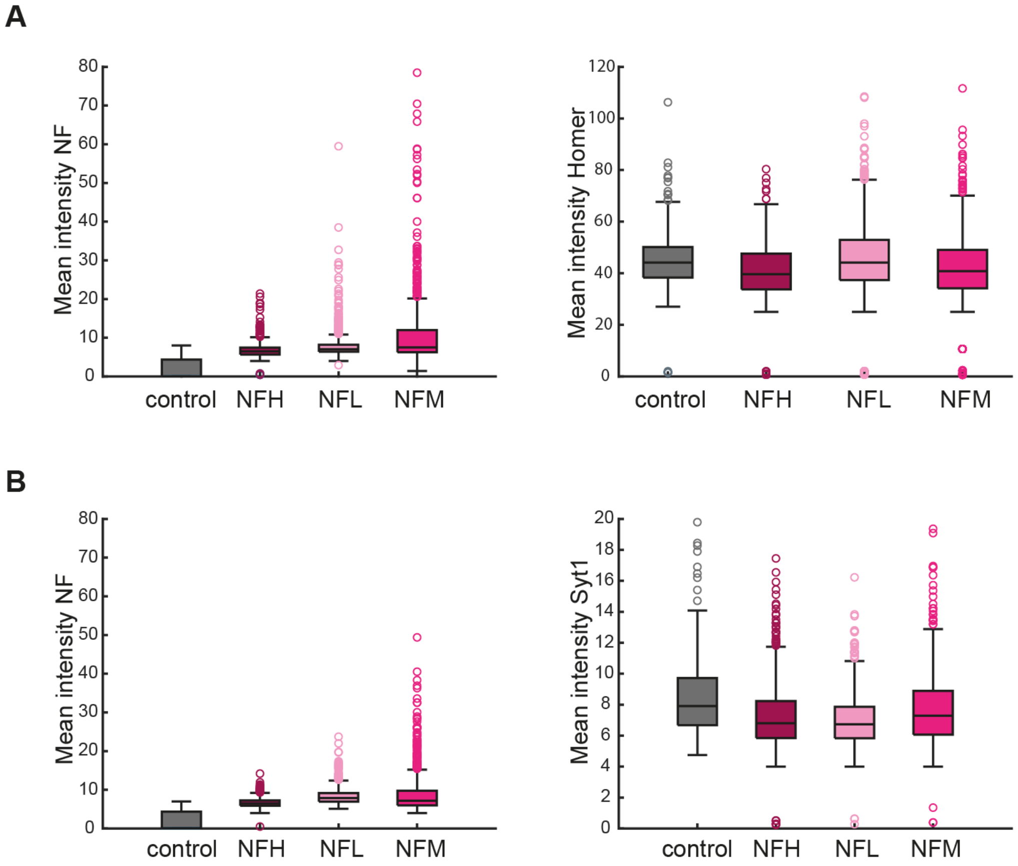

Figure S3. Intensity quantification of NF and synaptic labeling at manually segmented postsynapses in dual color STED images. (A) Mean intensity of NF (left) and homer (right) in manually segmented spines from 6 independent experiments. (B) Mean intensity of NF (left) and Syt1 (right) in manually segmented spines from 3 independent experiments. Controls are samples labeled without primary NF antibodies. Box-plots represent the datapoints included between then 25th and 75th percentile, whiskers 1.5 the inter quartile range, and circles the outliers. Values represent mean of raw photon counts.

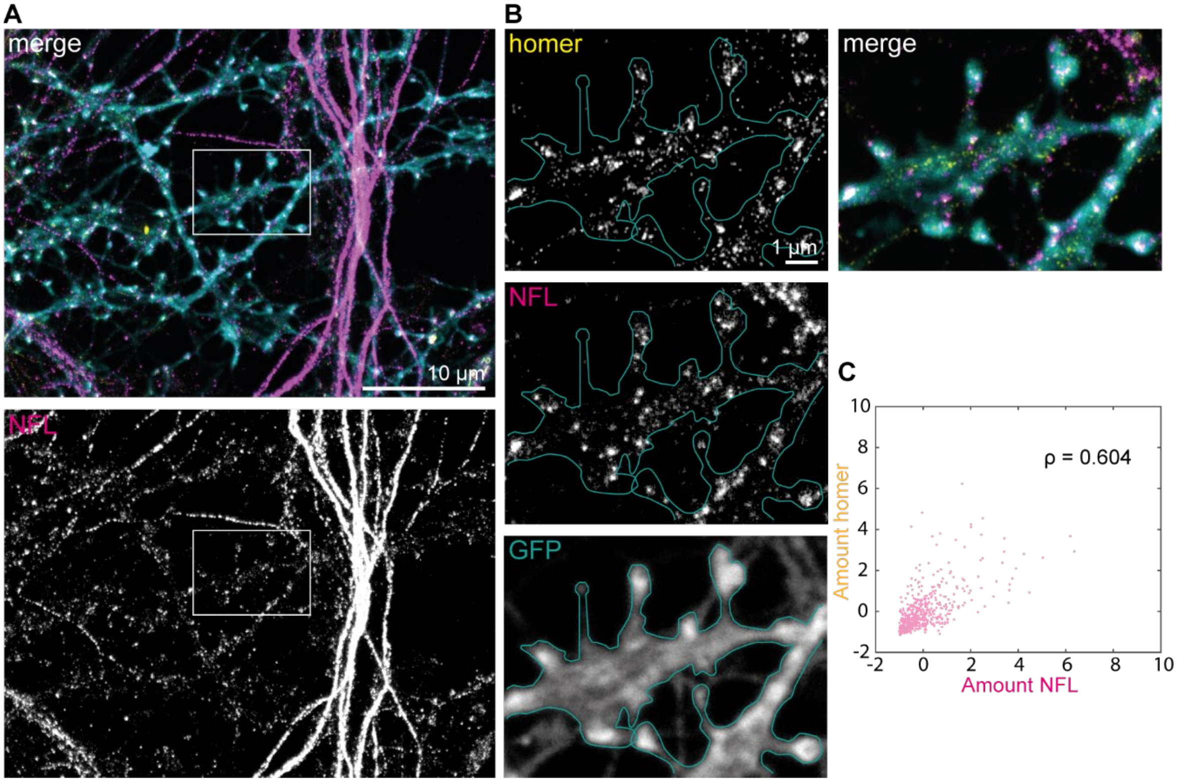

Figure S4. Correlation between NFL (mouse antibody) and homer. (A) Representative dual-color STED image of neurons with homer (yellow), NFL (mouse, magenta), and volume labeling (confocal, cyan). Corresponding single color channel representing NFL is shown in the lower panel. Samples were additionally stained with ankyrin G (rabbit, not shown). Scale bar: 10 µm. (B) Single color (left) and merge (right) close up images of the region indicated by the white box in (A). Cyan outline represents dendrites and spine shapes as determined by volume labeling. Scale bar: 1 µm. (C) Scatterplot of NFL (mouse antibody) versus homer protein amount (area × mean intensity). ϱ indicates Spearman's correlation coefficient with p value: 2.86×10^{-57}. Data was obtained from 563 manually segmented spines from 19 images and 2 independent neuronal cultures (21 DIV), standardized to the mean of each experiment. Amounts represent arbitrary units.
